# Effectiveness of Smoking Prevention Programs on the Knowledge, Attitudes, and Anti-Smoking Exposure Self-Efficacy among Non-Smoking Rural Seventh-Grade Students in Taiwan

**DOI:** 10.3390/ijerph19159767

**Published:** 2022-08-08

**Authors:** Su-Er Guo, Mei-Yen Chen, Chizimuzo Okoli, Yi-Fan Chiang

**Affiliations:** 1 Department of Nursing, College of Nursing, Chang Gung University of Science and Technology (CGUST), No. 2, Sec. W., Jiapu Rd., Puzi City 61363, Chiayi County, Taiwan; 2 Graduate Institute of Nursing, College of Nursing, Chang Gung University of Science and Technology (CGUST), No. 2, Sec. W., Jiapu Rd., Puzi City 61363, Chiayi County, Taiwan; 3 Chronic Diseases and Health Promotion Research Center, Chang Gung University of Science and Technology (CGUST), No. 2, Sec. W., Jiapu Rd., Puzi City 61363, Chiayi County, Taiwan; 4 Division of Pulmonary and Critical Care Medicine, Chang Gung Memorial Hospital, No. 6, Sec. West, Jiapu Rd., Puzi City 61363, Chiayi County, Taiwan; 5 Department of Safety Health and Environmental Engineering, Ming Chi University of Technology, No. 84, Gungjuan Rd., Taishan Dist., New Taipei City 24301, Taiwan; 6 College of Nursing, Chang Gung University of Science and Technology, No. 261, Wenhua 1st Rd., Guishan Dist., Taoyuan City 33303, Taiwan; 7 Tobacco Treatment and Prevention Division, Tobacco Policy Research Program, University of Kentucky College of Nursing, 315 College of Nursing Building, Lexington, KY 40536-0232, USA

**Keywords:** smoking prevention program, knowledge, attitude, secondhand smoke (SHS), self-efficacy

## Abstract

The disproportionate smoking prevalence among adolescents in rural Taiwan may be attributed to insufficient anti-smoking education. Increasing access to such education may help reduce initiation and promote smoking cessation in adolescents, particularly in rural areas. However, effects of these programs require verification. This study determined the effectiveness of a school-based prevention program in enhancing knowledge, attitudes, and anti-smoking exposure self-efficacy among seventh-grade non-smoking students. A quasi-experimental design with convenience sampling was employed, where participants included seventh graders from two junior high schools who completed a questionnaire 1–2 weeks before and after the intervention. Furthermore, the intervention group received four smoking and secondhand smoke (SHS) prevention classes, whereas the control group engaged in scheduled school activities. Knowledge on smoking (*B* = 4.38, *p* < 0.001) and SHS (*B* = 2.35, *p* < 0.001) were significantly greater in the intervention group. Moreover, the groups differed significantly in avoiding SHS exposure (*B* = 3.03, *p* = 0.031). Intervention modifications may be necessary to enhance the program’s effect on smoking exposure-related attitudes and self-efficacy. Additionally, cultural and other aspects (or “urban-rural gap”) might influence these results. Future randomized controlled trials should compare urban to rural adolescents, use longitudinal designs, and assess smoking initiation or cessation.

## 1. Introduction

Cigarette smoking exposure, both voluntary and involuntary, poses a serious threat to public health and smokers’ economic welfare [1]. The prevalence of Taiwanese cigarette smokers (ages ≥18) decreased from 26.3% in 1999 to 13.1% in 2020 [2]. Most adult smokers start smoking in adolescence; nearly 40% of Taiwanese adolescent smokers start smoking before the age of 14 [3]. Moreover, the prevalence of cigarette smoking among Taiwanese younger adolescents (aged 13–15) decreased from 7.8% to 3.0% between 1998 and 2019. Possible reasons for this decline are the 1997 Tobacco Hazards Prevention Act (THPA) and the 2009 THPA Amendment, which resulted in a strong governance against tobacco, a threat to the population’s health [4]. However, compared to 2018, the prevalence of smoking in the adolescent population increased slightly in 2019 (2.8% vs. 3.0%) [4]. Although not statistically significant, this was the first increase in 10 years since the implementation of the new regulations of the THPA in 2009. Tobacco use is the leading cause of preventable death worldwide. The World Health Organization (WHO) defines premature death due to tobacco use as a global epidemic. Thus, curbing cigarette smoking among Taiwanese adolescents is crucial to prevent the serious health risks associated with tobacco use (e.g., cardiovascular and respiratory diseases, cancer) [5].

Adolescents are most exposed to intense and consistent involuntary cigarette smoke or secondhand smoke (SHS) either at home or in public areas [6]. For adolescents, SHS exposure increases the risk of smoking initiation and the likelihood of cigarette dependence [7]. From 2008 to 2019, over 30% of adolescents reported SHS exposure in Taiwan [4]. Furthermore, SHS exposure causes cardiovascular and respiratory diseases in non-smokers, and any level of exposure is dangerous [5]. Globally, 50% of children are exposed to SHS at home, thus increasing their risk of respiratory illnesses and premature death [8,9].

Given that SHS exposure is related to both adoption of smoking behaviors and its detrimental health effects [10,11], interventions are needed to promote the avoidance of exposure among adolescents [12]. Accordingly, in 2009, Taiwan implemented the THPA Amendment, which entails the following: prohibiting smoking in individuals under the age of 18; forbidding smoking and cigarette advertisements in public buildings; increasing tobacco price; preventing cigarette sales to adolescents; and completely restricting smoking at all levels of school (up to high school) and in most enclosed public places. With the THPA, school-based anti-smoking education was promoted in most Taiwanese schools. However, Guo et al. [13] identified a higher percentage of cigarette-smoking adolescents in rural areas of Taiwan compared to the situation nationwide. This disproportionate smoking prevalence among adolescents may be attributed to insufficient implementation of anti-smoking education in rural Taiwan [4,14]. Furthermore, based on the Taiwan Global Youth Tobacco Survey, the most common reason for cigarette smoking initiation was “curiosity” [4]. Inadequate knowledge of SHS exposure’s impact may further contribute to the prevalence of smoking among adolescents [15]. Hence, increasing access to anti-smoking education may help promote an equitable distribution of THPA’s effects nationwide in reducing initiation and promoting the cessation of smoking among rural adolescents in Taiwan. However, first, the effects of such prevention programs on adolescents’ knowledge, attitudes, and anti-smoking exposure self-efficacy should be confirmed.

The correlation between psychosocial factors (attitude, knowledge, and self-efficacy) and cigarette smoking among adolescents has been demonstrated in several longitudinal and cross-sectional studies [5,16]. Adolescents are more likely to smoke when they exhibit a negative attitude toward anti-smoking, low confidence in their ability to avoid smoking, and a lack of knowledge of the health risks associated with smoking. Moreover, rural adolescents are more likely to possess permissive attitudes toward smoking than non-rural adolescents. Su et al. [17] examined the associations between attitude toward smoking and smoking behaviors in 2609 rural junior high school students and found that a positive attitude toward smoking was correlated to smoking behaviors. Therefore, altering rural adolescents’ attitudes is vital. Furthermore, Ma et al.’s [18] study found that compared with urban adolescents, rural adolescents, especially boys, exhibit poor anti-smoking self-efficacy and lower smoking refusal self-efficacy. Furthermore, although few studies have found an increase in the refusal skills of tobacco among adolescents [19], others have reported no change in anti-smoking self-efficacy following prevention programs [20]. Overall, numerous studies have found that rural residency is a risk factor for tobacco use among the youth.

Prior to smoking prevention intervention programs, the smoking rate was higher among boys in rural Taiwan; the important risk factors included poor academic achievement, smoking family members, negative attitude toward cigarette smoking, and anti-smoking self-efficacy [13]. In almost all adults, the mean age of smoking initiation was 15.3 years [5]. Moreover, smoking prevalence increases with age [5]. The rate is invariably higher among boys and correlated with low academic achievement among adolescents in all grades [21,22]. This trend can be observed among Taiwanese rural adolescents [13]. Low SES and the presence of smokers at home are associated with high smoking prevalence or early onset of smoking among the youth [5]. Furthermore, strict parental restrictions on smoking and close parental monitoring decrease adolescents’ smoking behavior [23,24]. The aforementioned factors may alter smoking behaviors among adolescents and influence smoking intervention programs’ effectiveness. Thus, the present study analyzed the between-group differences for the above relevant variables to evaluate the effectiveness of an intervention program, particularly, a smoking prevention program.

### Aim

As most adults begin smoking as teenagers, smoking prevention programs for adolescents are critical and could possibly be more effective than smoking cessation programs. Hence, this study aimed to evaluate a smoking prevention intervention’s effectiveness on the knowledge, attitudes, and anti-smoking exposure self-efficacy among rural junior high school students in Taiwan. Considering students from two rural junior high schools, we examined the effectiveness of the program in terms of the following aspects:

1. Improving the knowledge of the health risks of smoking and SHS exposure;
2. Promoting more negative attitudes toward smoking and SHS exposure;
3. Supporting anti-smoking and SHS exposure avoidance self-efficacy.

Smoking prevention intervention is crucial for addressing the increased risk of smoking among rural adolescents. Our study findings are significant for future research and have practical implications in programming and policy development for tobacco use control within rural school systems.

## 2. Materials and Methods

### 2.1. Study Design

A quasi-experiment design with convenience sampling was adopted to evaluate the effectiveness of a smoking prevention program on the knowledge, attitude, and anti-smoking exposure self-efficacy of seventh-grade students in rural Taiwan. Participants were assigned to intervention and control groups based on the school and were administered a questionnaire 1–2 weeks before (T1) and after (T2) the intervention. The intervention group received four smoking and SHS prevention classes within three months of a semester, while the control group engaged in scheduled school activities.

### 2.2. Participants

**Figure 1:**
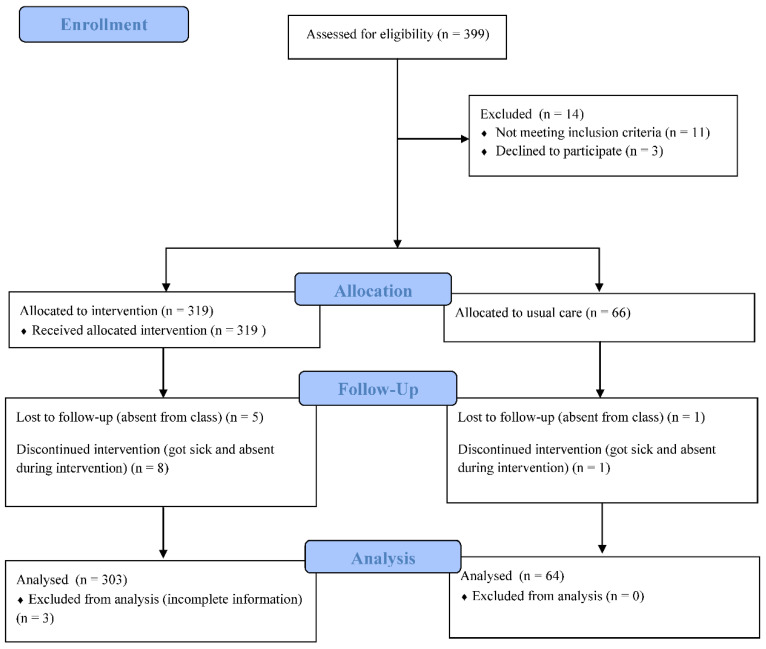
Flow Diagram of this study with Consolidated Standards of Reporting Trials (CONSORT).

Convenience sampling was employed to enroll all eligible seventh-grade students from two junior high schools with similar value teaching systems in rural and coastal areas in Taiwan. As inclusion criteria, eligible participants were required to be present at the baseline, display an ability to read and speak Chinese, and be willing to complete the questionnaires. Considering the exam and class schedules, one school allowed 13 classes of seventh-grade students to participate, among which some students declined to participate (*n* = 3), which served as the intervention group (*n* = 319), while the other school allowed two classes to participate, which served as the control group (*n* = 66). We excluded current smokers after intervention and data collection to avoid bias from different perspectives between smokers and non-smokers. Therefore, 367 students’ data were used for analysis after excluding those who provided incomplete data (*n* = 3), discontinued intervention (*n* = 9), or were lost during follow up (*n* = 6). Figure 1 presents a Consolidated Standards of Reporting Trials (CONSORT) flow diagram of this study.

### 2.3. Intervention

The intervention comprised four lessons (50 min each); the components of the intervention were developed based on the effective aspects of educational programs on smoking prevention [25,26] (see Table 1). During a semester period, the lessons were delivered in the classroom by trained health educators from Chang Gung University of Science and Technology and Chang Gung Memorial Hospital.

### 2.4. Process of Research

Prior to data collection, the Institutional Review Board of the relevant institution reviewed and approved the study procedure. Participants were informed of the purpose, benefits, and risks of the study and had the opportunity to ask questions. A signed consent form was obtained from those who agreed to participate in the study. The survey was anonymous to obtain reliable data and ensure confidentiality. At baseline and follow up, students completed a self-administered questionnaire in the classroom. Follow-up surveys were administered approximately eight months after the baseline for both groups. The classes of the same school groups were assessed on the same day.

### 2.5. Instruments

A structured questionnaire inquired about individual background characteristics, knowledge of smoking and SHS, SHS exposure avoidance, attitudes toward cigarette smoking and avoiding SHS, anti-smoking self-efficacy, and self-efficacy of SHS avoidance. The contents of each scale are described as follows.

#### 2.5.1. Background Characteristics

Background characteristics measured at baseline included age (in years), gender, parental education level, academic achievement, and perceived family income. Clinical factors included the perceived health status and exercise frequency in the last week, both in and outside the school. Furthermore, parents’ attitudes toward their children’s smoking and the number of family members who were current smokers were reported by the participants.

#### 2.5.2. Knowledge of Smoking and SHS

A 30-item scale developed by Kao [25] was used to assess participant knowledge of the health effects of smoking and the government’s tobacco control policy. The responses to the items can be either “True”, “False”, or “Do not know”; one point is assigned for each right answer. Possible scores range from 0 to 30, with higher scores indicating better knowledge. The scale’s content validity has been approved by experts. Based on Shi’s study, the scale-content validity index/average (SCVI/Ave) is 0.92, indicating good validity [27]. The Cronbach’s α of the scale is 0.82. In this study, the Kuder-Richardson 20 (KR-20) reliability of 0.86 indicated good reliability.

Furthermore, a 16-item questionnaire developed by Kurtz et al. [28] and modified by Wang et al. [29] was used to evaluate the knowledge of SHS and the side-effects of SHS exposure. The items can be responded with “True”, “False”, or “Do not know”, and the correct response receives one point. Possible scores range from 0 to 16, with higher scores indicating better knowledge of SHS risks. The KR-20 coefficient was 0.88 [29] and 0.87 in this study.

#### 2.5.3. Attitudes toward Cigarette Smoking and Avoiding SHS

A 13-item scale for measuring attitudes toward cigarette smoking was developed and modified by Tseng [30] (Cronbach’s α = 0.86). Each item is rated from 1 (strongly agree) to 5 (strongly disagree), while reverse items are coded oppositely. Possible scores range from 0 to 65, with higher scores indicating a more positive attitude toward cigarette smoking. In this study, the scale’s Cronbach’s α was 0.87.

Additionally, a 12-item questionnaire developed by Kurtz et al. [28] and modified by Li and Wang [31] was used to evaluate the participants’ attitude toward avoiding SHS. Each item is a feeling and belief statement related to SHS exposure. The 12 items are rated on a 5-point scale ranging from 1 (strongly disagree) to 5 (strongly agree), and negatively valenced items were reverse coded. Possible scores range from 12 to 60, with higher scores indicating more positive attitudes toward SHS avoidance. The Cronbach’s α was 0.86 [31] and 0.85 in this study.

#### 2.5.4. Avoidance of SHS

A nine-item scale developed by Martinelli [32] and modified by Wang et al. [29] was implemented to assess actions to avoid SHS exposure in different situations. The nine items are rated on a 4-point scale, ranging from 1 (not always) to 4 (yes, always); reverse items are coded oppositely. Possible scores range from 9 to 36, with higher scores indicating a higher number of actions performed to avoid SHS. The Cronbach’s α was 0.79 in the original study [29] and 0.69 in this study.

#### 2.5.5. Anti-Smoking Self-Efficacy and Self-Efficacy of SHS Avoidance

A 17-item questionnaire developed by Kao [25] was used to evaluate the participants’ confidence in their ability to resist smoking temptation in different situations. A 10-point scale (0 = no confidence, 10 = extreme confidence) is used to respond to the questionnaire. Possible scores range from 0 to 170, and higher scores indicate greater self-efficacy. Moreover, this scale’s content validity has been approved by experts in the field and SCVI/Ave is 0.92. Cronbach’s α was 0.93 in the original study and 0.91 in this study, indicating good reliability.

Furthermore, a 13-item scale of self-efficacy for SHS avoidance—developed by Martinelli et al. [33] and modified by Li and Wang [31]—was employed to assess the participants’ confidence in avoiding SHS exposure in different situations. A 5-point scale ranging from 1 (not at all confident) to 5 (extremely confident) is used to respond to the 13 items. Possible scores range from 13 to 65, with higher scores reflecting a better ability to avoid SHS exposure. The Cronbach’s α was 0.83 [31], while it reached 0.95 in this study.

### 2.6. Statistical Analyses

We compared participant characteristics between the control and intervention groups using the chi-square test for categorical variables or independent sample t-tests for continuous variables. The program’s intervention effects on the participants were assessed using the generalized estimating equation (GEE) model. Each GEE model included the main effect of the group (experimental group vs. control group), the main effect of time (posttest vs. pretest), a two-way interaction effect of the group by time, and the main effect(s) of possibly confounding variables (control variable). Group differences between changes in the pretest and posttest were verified if the two-way interaction effect was statistically significant. Data analysis was conducted using SPSS 22 (IBM SPSS, IBM Corp, Armonk, NY, USA).

## 3. Results

### 3.1. Participant Characteristics and Clinical Factors

The mean age of participants was 12.6 years (SD = 0.29), and 49.2% of the sample were women. Exposure to SHS (i.e., the number of smoking family members) was common, and the mean number of smoking family members was 0.98 (SD = 0.94). Most participants perceived no difference related to family income as compared to their peers, while 21.8% considered their family income to be poor. Furthermore, none of the participants’ parents supported their teenagers’ smoking behavior.

Table 2 presents the distribution of sociodemographic and clinical characteristics between the intervention and control groups. The participants in the control group self-reported being in a better health condition than those in the intervention group (52.4% vs. 33.6%). However, the mother’s education level was higher in the intervention group (65.5% vs. 51.6%). Moreover, there were no other differences between the groups related to the demographic and clinical factors. These two variables were adjusted in further GEE analysis.

### 3.2. Changes in Knowledge of Smoking-Related Health Risks and SHS Exposure

Table 3 shows the descriptive statistics of each outcome measure in the pretest and posttest for both groups. After the intervention, both scores on knowledge of smoking and SHS had significantly improved for the intervention group; in the control group, however, a significant decline in the knowledge of smoking was recorded. After adjusting for mother’s education and self-perceived health status in the GEE analysis (see Table 4), as compared to the control group, the improvements in the knowledge of smoking (*B* = 4.38, *p* < 0.001) and SHS (*B* = 2.35, *p* < 0.001) remained greater in the intervention group. Thus, the intervention appeared to have enhanced the intervention group’s knowledge of smoking and SHS, compared to the control group.

### 3.3. Changes in Attitudes toward Smoking and Avoiding SHS

After the intervention, no significant changes in the intervention group’s attitudes toward smoking and avoiding SHS were found. However, there was a significant decline in attitudes toward avoiding SHS exposure in the control group (see Table 3). In the GEE analysis (see Table 4), after adjusting for mother’s education and self-perceived health status, a significant difference was found between the groups regarding avoiding SHS exposure. Notably, a significant decline in the control group, which had lower scores than the intervention group, was recorded.

### 3.4. Changes in Anti-Smoking and SHS Exposure Avoidance Self-Efficacy

After the intervention, although no significant changes were found in SHS avoidance self-efficacy (see Table 3), significant declines in anti-smoking self-efficacy were identified in both intervention and control groups. In the GEE analysis (see Table 4), no differences were recorded between the intervention and control groups’ anti-smoking self-efficacy even after adjusting for the mother’s education and self-perceived health status. As the decline between pretest and posttest scores remained significant, how the intervention may have impacted anti-smoking self-efficacy was inconclusive.

**Figure 2:**
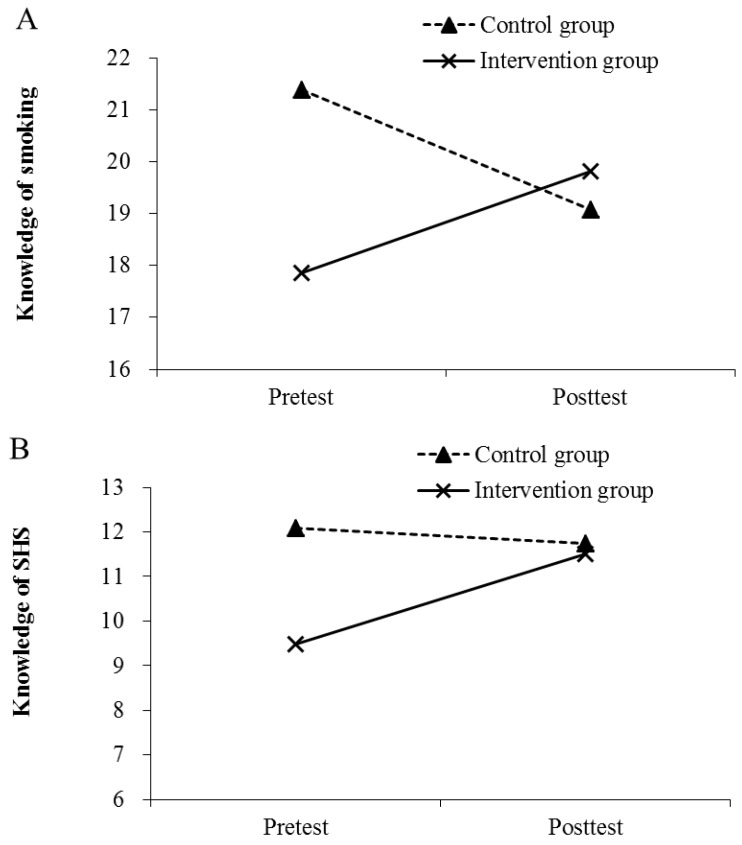

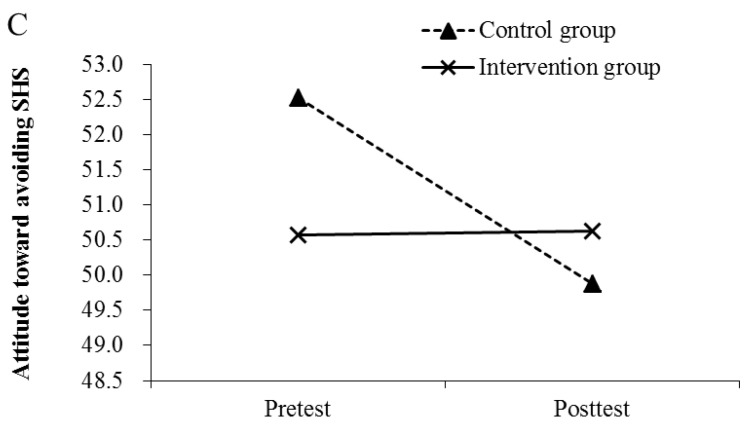
Mean score of (**A**) Knowledge of smoking, (**B**) Knowledge of SHS, and (**C**) Attitude toward avoiding SHS in the pretest and posttest for each group.

Figure 2 summarizes the changes in the mean scores of the three outcome measures (i.e., knowledge of smoking, knowledge of SHS exposure, and attitudes toward SHS avoidance), exhibiting improvement as a result of the intervention program.

## 4. Discussion

This study determined whether a school-based smoking prevention program could enhance SHS and smoking-related knowledge, attitudes, and anti-smoking self-efficacy among junior high school students. Although the intervention exhibited clear benefits for smoking prevention, smoking exposure-related knowledge, and SHS attitude, its impact on smoking-related attitudes or anti-smoking exposure self-efficacy was limited.

### 4.1. Effects of Smoking Prevention Programs on Knowledge and Attitudes

The school-based smoking prevention program significantly improved the knowledge of smoking and SHS exposure in the intervention group. Like our study, a cluster randomized controlled trial with 3444 students from 45 public secondary schools in Germany found an increase in smoking-related knowledge at six months and two years after a prevention program [19]. The authors identified a lower incidence of smoking and greater change in more critical attitudes toward the risks and disadvantages of smoking in the intervention group. However, studies examining the changes in smoking exposure-related knowledge following a prevention intervention are sparse. Most studies on SHS knowledge have used correlational designs and surveys to elucidate the relationship between SHS knowledge, attitudes, and behaviors [34,35]. Future studies may further examine longitudinal and practical outcomes (such as smoking initiation, trying smoking, or SHS avoidance behavior) in addition to changes in knowledge after delivering prevention interventions.

Furthermore, we identified that our intervention did not yield significant changes in smoking-related attitudes. Our finding contradicts a recent cluster randomized controlled trial conducted with 427 schoolchildren in Indonesia, which found an increase in anti-smoking attitudes following the delivery of a health education and an Islamic education program (as compared to the controls) [36]. Moreover, Isensee et al. [20] demonstrated an improvement in anti-smoking attitudes as a result of a prevention program in Germany. The differences between our findings and those of the above studies may be explained by the content and duration of smoking prevention programs. For example, Isensee et al.’s [19] program was more intensive, with a delivery of 14 units (90 min each) and two workshops (a total of 4–6 h). However, among urban adolescents in Taiwan, Lee et al. [37] conducted six smoking prevention courses within six months to enhance their knowledge and attitudes and found that this intensive program did not impact students’ smoking refusal behaviors. Likewise, in a critical review of reviews, Flay concluded that school-based smoking prevention programs can exhibit substantial long-term effects if they include at least 15 sessions and continued for multiple years [38]. Future studies, therefore, may assess the effects of the intensity, number of sessions, and duration of program delivery and content on participants’ attitudes.

Interestingly, although our intervention did not yield significant changes in smoking-related attitudes, it could maintain a positive attitude toward SHS avoidance in the intervention group, as compared to the control group. A previous study in Taiwan reported an increase in the avoidance behaviors of adolescents with an increase in their attitudes toward avoiding SHS exposure [29]. Therefore, as this attitude impacted the study’s findings (see Table 4), future studies may examine the motivation and exact behaviors of avoiding SHS exposure among rural adolescent populations in Taiwan experiencing high exposure.

### 4.2. Effects of Smoking Prevention Programs on Anti-Smoking Exposure Self-Efficacy

According to this analysis, school-based smoking prevention programs’ impact on anti-smoking self-efficacy has been lower than expected. This is an anomalous finding that may be explained by potential extraneous unknown factors within the school environment or social atmosphere. Likewise, as previously mentioned, adolescents experiencing SHS exposure at home [4] might believe that smoking is a normal behavior and should be allowed by people of authority, including their parents and relatives. Furthermore, adolescence is the most varying stage of life development. This period is often characterized by energy, impulsivity, rebelliousness, and self-centeredness. These traits can often precipitate deviant behavior. Moreover, adolescents’ social skills are underdeveloped, and thus, they often use smoking as a means to blend into their peer groups, applying self-righteous methods to find their social position [39]. Therefore, it is possible that even if they were taught to use refusal skills for anti-smoking with their peers, their self-efficacy would not be significantly changed. Furthermore, the lack of attractiveness in the design of program activities could be another reason for the lack of significant differences in students’ anti-smoking self-efficacy in the intervention group. Future studies may need to examine the effects of program content, activities, and different stages of adolescence on anti-smoking self-efficacy.

Noteworthily, a study examining the differential impacts of a school-based prevention program in Tibetan and Han adolescents found that while Tibetan students’ attitudes toward smoking became more positive, there was no change among Han students [40]. This finding may indicate some cultural or ethnic variations in the influence of school-based smoking prevention programs on the participants. As our study population was based in rural Taiwan, developing comparative analyses with other populations (such as urban students) might be helpful to determine potential cultural mediators in the efficacy of the program.

### 4.3. Strengths and Limitations

One of our study’s strengths is that the professional healthcare providers could deliver all four curriculum sessions completely and timely. Furthermore, we used scales with good validity and reliability and applied appropriate statistical analysis techniques (GEE) to evaluate the effects. However, several methodological limitations existed in this study. First, this study did not randomly assign intervention and control groups within the two included schools. Instead, we recruited a school as an intervention group and another one as a control group to avoid contamination within schools. Further, our results may be biased owing to the different sample sizes of the intervention and control schools in this study. Thus, extraneous factors related to the control school could have affected the main findings. For instance, the control school may have had different SHS exposure and smoking norms. Second, due to the administrative considerations of the intervention school, only four prevention courses were conducted, which may not have adequately impacted attitudes toward smoking and self-efficacy. Moreover, an order effect in the intervention of the four lessons might have impacted our actual intervention, and this problem should be considered in future studies. Finally, the analysis only focused on the immediate post-intervention effects and did not follow-up with the participants for an extended period. Long-term follow-up is important in demonstrating the intervention’s sustained impact. Future research should use longer-term follow-up time points and other smoking behavior variables (e.g., smoking initiation or cessation) to better evaluate such smoking prevention programs. To the best of our knowledge, despite these limitations, this quasi-experimental study is the first to evaluate a nurse-led smoking prevention program for adolescents in rural and coastal areas of Taiwan. Moreover, because different Asian cultures exhibit similar attitudes toward smoking, our findings provide insight into the development of future prevention programs and interventions in Taiwan, as well as results that are possibly close to those involving other Asian countries.

### 4.4. Clinical Practical Implications

Although this program did not improve anti-smoking attitudes and self-efficacy among adolescents, it improved knowledge on smoking and SHS and the attitude toward avoiding SHS exposure, and may have provided some preliminary evaluation data on the effectiveness of such programs in rural settings. Therefore, current interventions can be applied to rural adolescent populations. However, additional technical lessons and practice hours or workshops may be required to improve adolescent anti-smoking self-efficacy.

## 5. Conclusions

The smoking prevention program led to immediate improvements in the knowledge of smoking and smoking exposure as well as the attitude of avoiding SHS exposure among adolescents in Taiwan’s rural and coastal areas. However, the intervention might require modifications to enhance its impact on anti-smoking attitudes and self-efficacy. Thus, future randomized controlled trials should compare urban and rural adolescents, use longitudinal designs with longer timeframes, and assess smoking initiation or SHS avoidance behavior impacts. Such studies are important for supporting policies and guiding interventions for the prevention of the disproportionate tobacco-related disease burden among rural adolescents.

## Figures and Tables

**Table 1:** The detailed information of smoking prevention programs.

Topic	Contents	Material/Teaching Methods	Time
Information about cigarette smoking and its Health risks	History of tobacco Cigarettes components Health risks of cigarette smoking Smoking rates of Taiwan adolescents	PowerPoint Videos/Group discussion and feedback	50 min
Media awareness	Increase awareness of message delivered by media and cigarette advertising Tobacco Hazards Prevention Act	PowerPoint Learning sheets Videos/Discussion and feedback	50 min
Stress and coping & decision making skills	Dealing with pressure from peers and adults Improve the understanding of the decision-making process Learning refusal skills and resisting temptation to smoke	Power Point Learning sheets Video/Discussion and feedback	50 min
Smoke-free environments	Strengthening anti-smoking attitudes Strengthening the advantage of smoke-free environment Learning to create smoke-free environment	Power Point Learning sheets Video/Discussion and feedback	50 min

**Table 2:** Differences of demographic characteristics of participants between the experimental and control groups (*n* = 367).

Variable	Control, *n*(%)	Experimental, *n*(%)	χ^2^ (*p* Value)/*t* Test (*p* Value)
	64 (17.4)	303 (82.6)	
*Sociodemographic and background factors*			
**Sex**			0.02 (0.86)
Female	32 (50.0)	148 (49.0)	
Male	32 (50.0)	154 (51.0)	
**Mother’s education level**			4.40 (0.04) *
High school	33 (51.6)	192 (65.5)	
Less than high school	31 (48.4)	101 (34.5)	
**Father’s education level**			1.38 (0.24)
High school	29 (45.3)	157 (53.4)	
Less than high school	35 (54.7)	137 (46.6)	
**Perceived family economy status** (compared to his/her peers)			1.59 (0.45)
rich	16 (25.8)	77 (25.6)	
the same	29 (46.8)	162 (53.8)	
poor	17 (27.4)	62 (20.6)	
**Academic achievement**/academic performance #			1.53 (0.47)
high	23 (35.9)	113 (39.0)	
Medium	27 (42.2)	132 (45.5)	
Low	14 (21.9)	45 (15.5)	
**Perceived health** (compared to his/her peers)			7.99 (0.02) *
Good	33 (52.4)	101 (33.6)	
the same	19 (30.2)	122 (40.5)	
Poor	11 (17.5)	78 (25.9)	
*Social influences and environmental factors*			
**Parental disapproval of adolescent smoking**			0.08 (0.78)
strongly disapprove	58 (90.6)	271 (89.4)	
Disapprove	6 (9.4)	32 (10.6)	
approve	0	0	
**Age** (Mean ± SD) ^※^	12.59 ± 0.29	12.60 ± 0.30	−0.31 (0.76)
**the number of smoking family** (Mean ± SD) ^※^	1.11 ± 0.91	0.95 ± 0.94	1.26 (0.21)
**Exercise in school** (Mean ± SD) ^※^	3.39 ± 1.94	3.24 ± 1.74	0.60 (0.55)
**Exercise out school** (Mean ± SD) ^※^	3.61 ± 2.13	3.53 ± 2.02	0.27 (0.79)

Notes: * *p* < 0.05; # Academic achievement: high = top 40% of the class; medium = 41% to 80% of the class; low = bottom 20% of the class; ^※^ *t* test was used to compare the difference between two group.

**Table 3:** Descriptive statistics of each outcome measure in the pretest and posttest.

Variable	Control (*n* = 64)		*t*-Test	Experimental (*n* = 303)		*t*-Test
	Pretest	Posttest	*t*	Pretest	Posttest	*t*
Knowledge of smoking	21.4 ± 3.2	19.1 ± 5.8	−3.53 **	17.9 ± 5.7	19.8 ± 5.4	6.73 ***
Knowledge of SHS	12.1 ± 3.0	11.7 ± 3.7	−0.84	9.5 ± 4.3	11.5 ± 3.8	8.3 ***
Attitudes toward cigarette smoking	57.5 ± 9.6	55.9 ± 9.6	−1.05	56.8 ± 7.2	56.5 ± 7.2	−0.66
Attitude toward avoiding SHS	52.5 ± 7.9	49.9 ± 9.6	−2.27 *	50.6 ± 8.9	50.6 ± 8.2	0.28
Avoidance of SHS	28.7 ± 4.9	27.9 ± 4.4	−1.54	28.6 ± 4.7	28.4 ± 4.5	−0.28
Anti-smoking self-efficacy	140.7 ± 21.6	132.4 ± 24.9	−3.76 ***	137.8 ± 28.5	133.9 ± 28.6	−2.02 *
Self-efficacy of avoiding SHS	52.2 ± 12.1	50.8 ± 11.1	−0.92	50.3 ± 14.1	50.7 ± 11.7	0.37

Possible score of each scale: Knowledge of smoking range from 0 to 30, with higher scores indicating better knowledge; Knowledge of SHS range from 0 to 16, with higher scores indicating better knowledge of SHS risks; Attitudes toward cigarette smoking range from 0 to 65; Attitude toward avoiding SHS range from 12 to 60, with higher scores indicating more positive attitudes toward SHS avoidance; Avoidance of SHS range from 9 to 36; Anti-smoking self-efficacy range from 0 to 170; Self-efficacy of avoiding SHS range from 13 to 65. * *p* < 0.05, ** *p* < 0.01, *** *p*< 0.001.

**Table 4:** Effects of smoking prevention programs on knowledge, attitude, and self-efficacy (*n* = 367).

Parameter	Knowledge of Smoking		Knowledge of SHS		Attitudes toward Cigarette Smoking		Attitude toward Avoiding SHS		Avoidance of SHS		Anti-Smoking Self-Efficacy		Self-Efficacy of Avoiding SHS	
	*B*	*p*	*B*	*p*	*B*	*p*	*B*	*p*	*B*	*p*	*B*	*p*	*B*	*p*
Intercept	21.36 ***	<0.001	12.10 ***	<0.001	56.50 ***	<0.001	51.58 ***	<0.001	28.21 ***	<0.001	134.21 ***	<0.001	50.58 ***	<0.001
Time														
Posttest vs. Pretest	−2.40 ***	<0.001	−0.37	0.416	−1.68	0.295	−2.92 *	0.023	−1.03	0.084	−8.79 ***	<0.001	−1.25	0.475
Group														
Exp. vs. Con.	−3.70 ***	<0.001	−2.59 ***	<0.001	−0.41	0.758	−1.66	0.153	0.01	0.985	−2.28	0.472	−0.47	0.785
Interaction														
Group × Time	4.38 ***	<0.001	2.35 ***	<0.001	1.35	0.420	3.03 *	0.031	0.82	0.213	4.82	0.091	1.44	0.467

Exp. = experimental group; Con. = control group; SHS = Second Hand Smoke; Analysis was adjusted for mother’s education and self-perceived health status; * *p* < 0.05, *** *p* < 0.001.

## Data Availability

The data presented in this study are available on request from the corresponding author. The data are not publicly available due to the privacy of participants.
